# The Association between Low Muscle Mass and Hepatic Steatosis in Asymptomatic Population in Korea

**DOI:** 10.3390/life11080848

**Published:** 2021-08-19

**Authors:** Goh-Eun Chung, Hyo-Eun Park, Min-Joo Kim, Min-Sun Kwak, Jong-In Yang, Su-Jin Chung, Jeong-Yoon Yim, Ji-Won Yoon

**Affiliations:** 1 Departments of Gastroenterology and Hepatology, Internal Medicine, Seoul National University Hospital Healthcare System Gangnam Center, Seoul 06236, Korea; gohwom@snu.ac.kr (G.-E.C.); kms39@snuh.org (M.-S.K.); drmirinae@snuh.org (J.-I.Y.); medjsj@snuh.org (S.-J.C.); yjy@snuh.org (J.-Y.Y.); 2 Cardiology, Internal Medicine, Seoul National University Hospital Healthcare System Gangnam Center, Seoul 06236, Korea; 65529@snuh.org; 3 Endocrinology, Internal Medicine, Seoul National University Hospital Healthcare System Gangnam Center, Seoul 06236, Korea; 65986@snuh.org

**Keywords:** low muscle mass, hepatic steatosis, obesity

## Abstract

Background: An association between low muscle mass and nonalcoholic fatty liver disease (NAFLD) has been suggested. We investigated this relationship using controlled attenuation parameter (CAP). Methods: A retrospective cohort of subjects had liver FibroScan^®^ (Echosens, Paris, France) and bioelectrical impedance analyses during health screening exams. Low muscle mass was defined based on appendicular skeletal muscle mass/body weight ratios of one (class I) or two (class II) standard deviations below the sex-specific mean for healthy young adults. Results: Among 960 subjects (58.1 years; 67.4% male), 344 (45.8%, class I) and 110 (11.5%, class II) had low muscle mass. After adjusting for traditional metabolic risk factors, hepatic steatosis, defined as a CAP ≥ 248 dB/m, was associated with low muscle mass (class I, odds ratio (OR): 1.96, 95% confidence interval (CI): 1.38–2.78; class II, OR: 3.33, 95% CI: 1.77–6.26). A dose-dependent association between the grade of steatosis and low muscle mass was also found (class I, OR: 1.88, for CAP ≥ 248, <302; OR: 2.19, in CAP ≥ 302; class II, OR: 2.33, for CAP ≥ 248, <302; OR: 6.17, in CAP ≥ 302). High liver stiffness was also significantly associated with an increased risk of low muscle mass (class I, OR: 1.97, 95% CI: 1.31–2.95; class II, OR: 2.96, 95% CI: 1.51–5.78). Conclusion: Hepatic steatosis is independently associated with low muscle mass in a dose-dependent manner. The association between hepatic steatosis and low muscle mass suggests that particular attention should be given to subjects with NAFLD for an adequate assessment of muscle mass.

## 1. Introduction

Nonalcoholic fatty liver disease (NAFLD) is the most common type of liver disease, with a prevalence of 25% globally and 27% in Asia [1]. NAFLD is closely related to various metabolic conditions, such as visceral obesity, type 2 diabetes, and cardiovascular disease, and thus has been considered a hepatic manifestation of metabolic syndrome [2]. The gold standard for the diagnosis of NAFLD is liver biopsy, but due to its invasiveness and possible sampling error, the use of liver biopsy in clinical practice is extremely limited in asymptomatic individuals without overt liver disease. Instead, ultrasonography is recommended as the first-line modality for fatty liver evaluation [3]. However, the innate limitations of ultrasonographic assessments, such as interpersonal variability and inaccuracy in diagnosing early-stage steatosis, restrict their widespread use [3,4]. Controlled attenuation parameter (CAP) during transient elastography with FibroScan^®^ can be used to estimate hepatic steatosis with high sensitivity, allowing the early and noninvasive detection of NAFLD at subclinical stages [5,6,7]. Both the liver stiffness measurement (LSM) and CAP show a good correlation with pathologic findings in patients with NAFLD [8].

Sarcopenia, which is an age-related decline in skeletal muscle mass and strength with or without a reduction in physical performance [9] has been associated with increases in metabolic and cardiovascular diseases, disability, and mortality [10,11,12]. Growing evidence suggests a possible association between NAFLD and sarcopenia, with the same primary pathophysiology as insulin resistance [13,14]. In a recent meta-analysis, both the risk of NAFLD and the progression of NAFLD-related fibrosis were higher in subjects with sarcopenia than in those without, as assessed by ultrasonography [15,16,17,18].

In the current study, we used FibroScan to obtain an objective value for hepatic steatosis and fibrosis. We evaluated the association between low muscle mass and hepatic steatosis in the general Korean population without overt liver disease.

## 2. Methods

### 2.1. Study Population

We used a previously conducted retrospective study population [19]. Briefly, subjects who underwent bioelectrical analyses and coronary calcium scoring computed tomography at the Seoul National University Hospital Healthcare System Gangnam Center in 2017–2018 were included, and we added subjects for 2019–2020. Among them, we included subjects who underwent FibroScan. Most subjects were asymptomatic and underwent voluntary general health check-up exams, while others were sent by their employers. Initially, a total of 1428 subjects were enrolled. We excluded subjects who had a potential risk of chronic liver disease; 36 were pos itive for hepatitis B virus, 12 were positive for the hepatitis C vi rus, and 167 had significant alcohol intake (>20 g/day for males and >10 g/day for females) [20]. Additionally, 290 subjects were excluded due to missing information. Finally, 923 subjects were included in the final analysis.

The study protocol followed the guidelines of the Declaration of Helsinki of 1975, as revised in 1983. The protocol was approved by the Institutional Review Board of Seoul National University Hospital (No. 2006-024-1130). Informed consent was waived by the board since researchers accessed and analyzed only anonymized data.

### 2.2. Measurements of Clinical and Laboratory Parameters

Data regarding past medical history, comorbidities, and medications were obtained using subject-reported questionnaires. Blood pressure was measured at least twice, and mean values of the measurements were recorded. Hypertension was defined as a blood pressure greater than or equal to 140/90 mmHg or a history of receiving antihypertensive medications, and diabetes was defined as a fasting blood glucose level greater than or equal to 126 mg/dL, a glycated hemoglobin level greater than or equal to 6.5%, or a history of receiving glucose-lowering agents. Subjects taking lipid-lowering agents or having a total cholesterol level greater than or equal to 240 mg/dL were categorized as having hypercholesterolemia [21].

All blood samples were collected after a 12-hour overnight fast. Laboratory tests included levels of serum alanine aminotransferase (ALT), aspartate aminotransferase, total cholesterol, triglycerides, high-density lipoprotein (HDL) cholesterol, fasting glucose, creatinine, and high-sensitivity C-reactive protein (hs-CRP). All tests were performed using standard laboratory methods.

### 2.3. Anthropometric Measurements

Body weight and height were measured using a digital scale, and body mass index (BMI) was calculated by dividing the weight (kg) by the squared value of the height (m^2^). A tape was used to measure the waist circumference at the midpoint between the lower costal margin and anterior superior iliac crest. To assess body composition, bioelectrical impedance analysis (BIA) was performed using an InBody 720 Body Composition Analyzer (InBody Co., Ltd., Seoul, Korea), as described previously [22]. During this test, subjects remained in a standing position for 5 to 10 minutes with their legs slightly separated and their arms slightly abducted from the trunk. They were instructed to grasp the handles of the analyzer so that each extremity contacted the electrodes. Multi-frequency measurements of impedance for each segment (i.e., the trunk and four extremities) were obtained and used to estimate the appendicular skeletal muscle mass (ASM).

### 2.4. Definitions of Low Muscle Mass and Obesity 

ASM (kg) was calculated as the sum of the lean muscle mass in all four extremities. ASM% was calculated as ASM/weight (kg) * 100, as modified from Janssen et al. [23] Low muscle mass was defined according to ASM%. Class I low muscle mass was defined as more than one standard deviation (SD) and less than 2 SD below the sex-specific mean for healthy young adults, and class II low muscle mass was defined as 2 SDs below the sex-specific mean for healthy young adults, according to the nationwide health examinations of the Korean population (ASM% < 32.2 in men and <25.6 in women for class I; <29.1 in men and <23.0 in women for class II) [22,24].

### 2.5. CAP and Liver Stiffness Measurement

CAP and LSM were obtained by a FibroScan (Echosens, Paris, France) using an M (standard probe–transducer frequency 3.5 MHz) or XL probe (transducer frequency 2.5 MHz). XL probe was used for all patients with BMI of ≥30.0 kg/m^2^ [25,26]. The procedure was performed by an experienced investigator who was blinded to the clinical information, as described previously [27]. Briefly, the patient was placed in the dorsal decubitus position with the right arm maximally abducted. A FibroScan was performed on the right lobe of the liver through the intercostal spaces. The values of LSM were expressed as the median kilopascal (kPa) value, and the median CAP score was expressed in dB/m values. LSM values were considered reliable if 10 valid measurements were obtained and the interquartile range/median of the measurements was less than 0.3 or when the median liver stiffness value was less than 7.1 kPa [28]. All patients with 10 valid measurements were included in the analysis. In this study, CAP values of 248 dB/m was used to define hepatic steatosis [7]. For evaluation of marked hepatic steatosis, we used another CAP value of 302 dB/m [8,29,30].

### 2.6. Statistical Analysis

Continuous variables were expressed as the mean ± SD for normally distributed continuous variables; categorical variables were expressed as numbers and percentages. Log transformations were performed for non-normally distributed variables. Comparisons of continuous variables between groups were performed using the Student’s *t* test or analysis of variance, and categorical variables were compared using the chi-square test or Fisher’s exact test. LSM tertile 1 represented the lowest values (i.e., T1 < 3.4 kPa, T2 = 3.4–4.2 kPa, and T3 ≥ 4.3 kPa). To evaluate the parameters associated with low muscle mass, univariate and multinominal logistic analyses were performed. In the multinominal logistic analysis, age, sex, presence of hypertension, diabetes, and hypercholesterolemia; BMI values; and levels of triglycerides, HDL, ALT, and hs-CRP were adjusted as covariates. All statistical analyses were performed using SPSS 22.0 software (SPSS Inc., Chicago, IL, USA) and R version 3.2.3 (The R Foundation for Statistical Computing, Vienna, Austria, http://www.Rproject.org (accessed on 24 February 2021). *p* values less than 0.05 were considered statistically significant.

## 3. Results

### 3.1. Clinical Characteristics of the Study Population 

The mean age of our study population was 58 ± 10 years, and 67.4% of the subjects were male. Among the 960 subjects, 344 (45.8%, class I) and 110 (11.5%, class II) had low muscle mass. Clinical characteristics according to muscle mass are summarized in Table 1. Compared with the control group, individuals with low muscle mass were older, more frequently male, and had a higher BMI, waist circumference, and systolic blood pressure (*p* < 0.05). Serum ALT, total cholesterol, triglycerides, fasting blood glucose, and hs-CRP levels were also higher in individuals with low muscle mass than in the control group. Subjects with a low muscle mass also had a higher prevalence of higher CAP values and tertiles of LSM (*p* < 0.001).

### 3.2. Parameters Associated with Low Muscle Mass

Table 2 shows the association of each parameter with class I low muscle mass according to the univariate logistic regression analysis. Age; male sex; presence of hypertension, diabetes, and hypercholesterolemia; BMI value and levels of triglycerides, HDL-cholesterol, ALT, and hs-CRP were significantly associated with low muscle mass (*p* < 0.05). Class I low muscle mass was associated the CAP ≥ 248 dB/m and CAP ≥ 302 dB/m (odds ratio (OR): 3.91, 95% confidence interval (CI): 2.97–5.15 and OR: 3.55, 95% CI: 2.51–5.03, respectively). LSM also correlated with low muscle mass (OR: 1.43, 95% CI: 1.27–1.31, *p* < 0.001); when tertiles were evaluated, a higher tertile of LSM was associated with low muscle mass in a dose-dependent manner compared to the lowest tertile (OR: 1.49, 95% CI: 1.08–2.07 for T2; OR: 3.22, 95% CI: 2.31–4.49 for T3 vs. T1). When we evaluated class II low muscle mass as an outcome variable, the results were similar except in the presence of diabetes and hypercholesterolemia (Supplementary Table S1).

### 3.3. Association between NAFLD and Low Muscle Mass

Multinominal logistic analysis was performed to evaluate the association between hepatic steatosis and low muscle mass. When adjusting for age and sex, a high CAP value greater than or equal to 248 dB/m, was associated with low muscle mass (class I, OR: 3.09, 95% CI: 2.28–4.17; class II, OR: 7.24, 95% CI: 4.19–12.50). When we further adjusted for the presence of hypertension, BMI, and levels of triglycerides, fasting glucose, cholesterol, triglyceride, HDL-cholesterol, ALT, and hs-CRP, CAP ≥ 248 dB/m was significantly associated with low muscle mass (class I, OR: 1.96, 95% CI: 1.38–2.78; class II, OR: 3.33, 95% CI: 1.77–6.26, Table 3). To evaluate the association between the grade of hepatic steatosis and low muscle mass, we used two cut-off CAP values, as described above. In the multivariate analysis, there was a dose-dependent relationship between the grade of steatosis and low muscle mass (class I, OR: 1.88, 95% CI: 1.29–2.73 for CAP ≥ 248 but <302; OR: 2.19, 95% CI: 1.35–3.58 in CAP ≥ 302, and class II, OR: 2.33, 95% CI: 1.18–4.60 for CAP ≥ 248 but <302; OR: 6.17, 95% CI: 2.93–13.0 in CAP ≥ 302).

The association between LSM and low muscle mass was evaluated using the same multivariate model. The highest tertile of LSM showed a significant association with an increased risk of low muscle mass (class I, OR: 1.97, 95% CI: 1.31–2.95, *p* = 0.001; class II, OR: 2.96, 95% CI: 1.51–5.78, *p* = 0.002). 

### 3.4. Stratified Analysis According to Obesity

Since obesity is both a well-known confounder and significant risk factor for low muscle mass, we stratified the study population according to the presence of obesity (BMI > 25) and re-evaluated the association between hepatic steatosis and low muscle mass (class I). In the multivariate analysis after adjusting for age; sex; presence of hypertension, diabetes, and hypercholesterolemia; BMI value; and levels of triglycerides, HDL-cholesterol, ALT, and hs-CRP, the increased risk of low muscle mass in subjects with NAFLD was significant in both obese and nonobese patients (OR: 2.22, 95% CI: 1.46–3.37 vs. OR: 2.04, 95% CI: 1.10–3.78, respectively). When evaluated according to the grade of steatosis, a dose-dependent relationship persisted in both obese and nonobese patients (OR: 1.74 in CAP ≥ 248 but <302 and OR: 2.90 in CAP ≥ 302 vs. OR: 1.34 in CAP ≥ 248 but <302 and OR: 2.95 in CAP ≥ 302). A significant association between LSM and low muscle mass was found in both obese and nonobese patients (OR: 2.04, 95% CI: 1.25–3.32 vs. OR: 2.23, 95% CI: 1.12–4.45, Supplementary Table S2). 

## 4. Discussion

In this study, we determined the association between low muscle mass and hepatic steatosis, which is defined as a CAP value greater than or equal to 248 dB/m using a FibroScan. Hepatic steatosis was independently associated with low muscle mass, even after adjusting for traditional risk factors of metabolic disease in a Korean population that had undergone routine health screenings. There was a dose-dependent relationship between the grade of steatosis and low muscle mass. In addition, a high LSM exhibited a significant association with an increased risk of low muscle mass.

Since muscle loss is closely linked to insulin resistance and systemic inflammation, which also play essential roles in the pathogenesis of NAFLD, previous studies have demonstrated that low muscle mass or sarcopenia may affect the development of NAFLD [31]. A large study performed in Korea showed that sarcopenia was associated with an increased risk of NAFLD independent of obesity or insulin resistance [32]. However, the diagnoses of NAFLD based on fatty liver prediction models were limited in that study. A recent study reported that low muscle mass and strength were independently associated with ultrasonography-diagnosed NAFLD [33]. In the previous study, the cut-off values for low muscle mass were 28.6% for men and 24.1% for women, one SD below the sex-specific mean for the young reference population. These cut-off values differed from those used in our study (<32.2 in men and <25.6 in women for class I and <29.1 in men and <23.0 in women for class II), which might reflect differences in the methods used for NAFLD detection and the heterogeneity of the study population. Consistent with our study, the definition of low muscle mass varied less than 1 SD [34] or 2 SD below the sex-specific mean for the young reference group [24]. Hashimoto et al. [35] reported that the skeletal muscle index was negatively associated with hepatic steatosis, defined as a CAP value greater than 237.8 dB/m, in a small number of Japanese patients with type 2 diabetes. Taken together, in most previous studies, NAFLD was set as an outcome variable in individuals with or without sarcopenia or low muscle mass. Nevertheless, a causal relationship between NAFLD and low muscle mass is not yet clear. In this study, we set two cut-off values for low muscle mass as outcomes in subjects with or without NAFLD to determine whether NAFLD could be identified as a risk factor for low muscle mass. 

Because NAFLD is considered a hepatic manifestation of metabolic syndrome and has been associated with cardiometabolic diseases, its usefulness is not limited to just liver diagnostics. Previously, an association between CAP-defined NAFLD and increased arterial stiffness [36] or coronary artery plaques [21] has been reported, suggesting the role of FibroScan results in the risk stratification of patients with NAFLD.

Previous studies have found low muscle mass to be an independent risk factor for advanced fibrosis as assessed by NAFLD fibrosis score and the Fibrosis-4 index in a Korean population that had undergone routine health screenings [16]. In a prospective biopsy-confirmed cohort, low muscle mass was associated with significant fibrosis and advanced NAFLD [37]. Our results are consistent with previous findings that show a significant association between low muscle mass and LSM, which is a noninvasive, clinically useful method to assess the severity of hepatic fibrosis in patients with NAFLD [38,39].

The loss of muscle mass or strength is a complex process that involves changes in neuromuscular function, physical activity, levels of inflammatory cytokines, dietary intake, and levels of hormones, such as insulin, growth factor, and vitamin D [40]. The possible mechanisms responsible for the association between NAFLD and low muscle mass could be related to obesity, insulin resistance, chronic inflammation, and the signaling of hepatokines and myokines [14]. First, obesity, which is one of the main pathological drivers of NAFLD, is associated with lower physical function, including poor muscle quality and low fat-free mass [41,42]. Obesity causes ectopic fat accumulation in liver and skeletal muscle, stimulating muscles to produce myostatin and cytokines. These factors cause lipotoxicity, which synergistically leads to additional loss of muscle mass [43]. Further, the activation of adipocytes in obesity promotes proinflammatory processes and induces muscle atrophy [44]. Second, insulin resistance, the main characteristic of NAFLD, leads to the accumulation of triglycerides in muscle and exacerbates proteolysis, leading to muscle depletion [45]. Finally, chronic inflammation during liver injury [46] stimulates proinflammatory cytokines, such as tumor necrosis factor-alpha and interleukin-6, which may result in the deterioration of muscle mass [47].

Interestingly, the usefulness of CAP and LSM was found in both nonobese and obese subjects, with an increased risk of class I low muscle mass more prominent in obese patients. While currently there is no clear mechanism to explain our findings, obesity itself is a potent contributor to low muscle mass, which may aggravate both low muscle and NAFLD, so this vicious cycle may exert synergistic detrimental effects.

## 5. Limitations

Our study has some limitations. First, because the study was conducted as a retrospective observational study, we could not infer causal relationships. Second, BIA is not the gold standard method to evaluate muscle mass; however, it is a very useful and practical tool for screening purposes to evaluate muscle and fat mass, especially at health screening centers and primary clinics. In addition, it has been reported that muscle fat content, but not muscle mass, is strongly and independently associated with NASH [48]. However, we could not evaluate this factor because there was no data regarding imaging and histology. Third, although the results of a meta-analysis suggested an optimal cut-off value for distinguishing normal from hepatic steatosis [7], ideal cut-off CAP values for detecting and grading steatosis have not yet been established [49]; thus, accurate detecting and grading of hepatic steatosis using CAP value is limited in this study. However, the main purpose of this study was the early detection of NAFLD using CAP, rather than the accuracy of the CAP values, in potentially at-risk patients with low muscle mass. Although LSM using transient elastography is used for detection of significant fibrosis in patient with clinically relevant symptom, significant fibrosis with LSM ≥ 7 kPa was present in only 2.1% (*n* = 20) of our study; thus, we analyzed using LSM tertiles. However, since low LSM values do not separate the minor stages of fibrosis [50], there may be a possibility of over-interpretation of tertiles of LSM in those without signs of clinically significant fibrosis. Fourth, because we could not obtain information regarding muscle strength (e.g., grip strength) or physical performance, we could not evaluate sarcopenia as an outcome variable. Lastly, we could not rule out other chronic liver diseases such as hemochromatosis, autoimmune hepatitis, Wilson diseases, etc., and since not all health checkup examinees have undergone Fibroscan exams our institution, there may be a selection bias because we included only a subset of the total health screenings that underwent related exams. The results of our study, therefore, should be interpreted carefully. 

## 6. Conclusions

CAP-defined NAFLD is independently associated with low muscle mass in a dose-dependent manner. The association between NAFLD and sarcopenia suggests that careful attention should be given to subjects with NAFLD for risk stratification and appropriate assessments regarding muscle mass.

## Figures and Tables

**Table 1:** Comparison of baseline characteristics according to low muscle mass.

	Control (N = 506)	Low Muscle Mass I (N = 344)	Low Muscle Mass II (N = 110)	*p*-Value
Age (years)	57.7 ± 9.4	59.3 ± 9.7	60.2 ± 11.3	0.013
Male, *n* (%)	279 (55.1)	257 (74.7)	89 (80.9)	<0.001
Current smoking, *n* (%)	103 (20.4)	70 (20.3)	23 (20.9)	0.991
BMI (kg/m^2^)	22.7 ± 2.3	25.6 ± 2.6	28.8 ± 4.1	<0.001
BMI ≥ 25 (kg/m^2^), *n* (%)	84 (16.6)	200 (58.1)	94 (85.5)	<0.001
Waist circumference (cm)	84.2 ± 7.2	92.2 ± 7.4	100.0 ± 9.9	<0.001
ASM/weight, %	31.3 ± 3.3	29.2 ± 2.8	26.4 ± 2.8	<0.001
Systolic blood pressure, mmHg	121.4 ± 16.0	124.4 ± 15.8	125.4 ± 15.7	0.006
Diastolic blood pressure, mmHg	80.6 ± 10.6	81.7 ± 11.1	82.4 ± 10.7	0.191
*Comorbidities*				
Diabetes mellitus, n (%)	47 (9.3)	63 (18.3)	19 (17.8)	<0.001
Hypertension, n (%)	124 (24.5)	127 (36.9)	48 (43.6)	<0.001
Hypercholesterolemia, n (%)	126 (24.9)	122 (35.5)	37 (33.6)	0.003
*Laboratory parameters*				
AST (IU/L) ^+^	25 (20–30)	26 (21–33)	27 (21–32)	0.329
ALT (IU/L) ^+^	22 (16–31)	26 (19–38)	27 (20–44)	<0.001
Cholesterol (mg/dL)	193.7 ± 39.8	185.2 ± 41.5	200.8 ± 45.7	0.001
Triglyceride (mg/dL) ^+^	86 (65–130)	112 (82–158)	132 (90–193)	<0.001
HDL-cholesterol (mg/dL)	56.9 ± 15.4	51.4 ± 13.0	50.4 ± 13.7	<0.001
Fasting glucose (mg/dL)	103.1 ± 19.8	109.9 ± 24.5	112.8 ± 27.5	<0.001
Hemoglobin A1c, %	5.8 ± 0.7	6.0 ± 0.9	6.1 ± 0.9	<0.001
Creatinine (mg/dL)	0.83 ± 0.2	0.87 ± 0.2	0.86 ± 0.2	0.010
HS-CRP (mg/dL)	0.10 ± 0.3	0.16 ± 0.4	0.28 ± 0.6	<0.001
*Transient elastography*				
CAP, dB/m ^+^	238 (204–268)	271 (236–303)	299 (263–329)	<0.001
CAP ≥ 248	196 (38.7)	232 (67.4)	92 (83.6)	<0.001
CAP ≥ 302	59 (11.7)	87 (25.3)	53 (48.2)	<0.001
Liver stiffness measurement, kPa ^+^	3.5 (3.0–4.2)	3.9 (3.3–4.8)	4.3 (3.5–5.0)	0.001
Tertile 1 (<3.4)	204 (40.3)	92 (26.7)	17 (15.5)	<0.001
Tertile 2 (3.4–4.2)	182 (36.0)	108 (31.4)	38 (34.5)	
Tertile 3 (≥4.3)	120 (23.7)	144 (41.9)	55 (50.0)	

Data are shown as the mean ± SD. ^+^ median (interquartile range). *p*-value for analysis of variance. ASM, appendicular muscle mass; BMI, body mass index; AST, aspartate aminotransferase; ALT, alanine aminotransferase; CAP, controlled attenuation parameter; HDL, high-density lipoprotein; HS-CRP, high sensitivity C-reactive protein.

**Table 2:** Parameters associated with low muscle mass (class I).

Variables	Odds Ratio	95% Confidence Interval	*p*-Value *
Age, years	1.02	1.01–1.03	0.005
Male	2.59	1.96–3.44	<0.001
Hypertension	2.02	1.52–2.68	<0.001
Diabetes mellitus	2.15	1.45–3.19	<0.001
Hypercholesterolemia	1.58	1.19–2.10	0.002
Current smoking	1.02	0.74–1.40	0.919
Body mass index, kg/m^2^	1.71	1.59–1.84	<0.001
Fasting glucose, mg/dL	1.02	1.01–1.02	<0.001
Total cholesterol, mg/dL	1.00	0.99–1.00	0.113
Triglyceride, mg/dL ^+^	2.64	2.02–3.46	<0.001
HDL cholesterol, mg/dL	0.97	0.96–0.98	<0.001
AST, IU/L ^+^	1.47	1.05–2.06	0.025
ALT, IU/L ^+^	2.07	1.65–2.68	<0.001
HS-CRP (mg/dL)	3.07	1.56–6.03	0.001
CAP, dB/m	1.02	1.01–1.02	<0.001
CAP ≥ 248 dB/m	3.91	2.97–5.15	<0.001
CAP ≥ 302 dB/m	3.55	2.51–5.03	<0.001
LSM, kPa	1.43	1.27–1.61	<0.001
LSM, Tertile 1st	1 (reference)		<0.001
Tertile 2nd	1.49	1.08–2.07	0.015
Tertile 3rd	3.22	2.31–4.49	<0.001

ALT, alanine aminotransferase; AST, aspartate aminotransferase; CAP, controlled attenuation parameter; HDL, high-density lipoprotein; HS-CRP, high sensitivity C-reactive protein; LSM, liver stiffness measurement; * compared to normal muscle mass as reference; ^+^ log transformed.

**Table 3:** Multinominal logistic analysis for the association between hepatic steatosis and low muscle mass.

	Low Muscle Mass I				Low Muscle Mass II			
	Age and Sex Adjusted		Multivariate Model		Age and Sex Adjusted		Multivariate Model	
	OR (95% CI)	*p*-Value	OR (95% CI)	*p*-Value	OR (95% CI)	*p*-Value	OR (95% CI)	*p*-Value
CAP < 248 dB/m	1 (reference)		1 (reference)		1 (reference)	<0.001 *	1 (reference)	<0.001 *
CAP ≥ 248 dB/m	3.09 (2.28–4.17)	<0.001	1.96 (1.38–2.78)	<0.001	7.24 (4.19–12.50)	<0.001	3.33 (1.77–6.26)	<0.001
CAP Grade								
CAP < 248 dB/m	1 (reference)		1 (reference)		1 (reference)	<0.001 *	1 (reference)	<0.001 *
CAP ≥ 248, <302 dB/m	2.68 (1.93–3.74)	<0.001	1.88 (1.29–2.73)	0.001	4.14 (2.26–7.61)	<0.001	2.33 (1.18–4.60)	0.015
CAP ≥ 302 dB/m	4.10 (2.71–6.22)	<0.001	2.19 (1.35–3.58)	0.002	15.4 (8.24–28.7)	<0.001	6.17 (2.93–13.0)	<0.001
Liver stiffness measurement								
Tertile 1st	1 (reference)		1 (reference)		1 (reference)	<0.001 *	1 (reference)	0.002 *
Tertile 2nd	1.30 (0.91–1.86)	0.151	1.11 (0.75–1.65)	0.610	2.26 (1.22–4.20)	0.010	1.54 (0.77–3.06)	0.221
Tertile 3rd	2.51 (1.75–3.61)	<0.001	1.97 (1.31–2.95)	0.001	4.75 (2.60–8.67)	<0.001	2.96 (1.51–5.78)	0.002

ALT, alanine aminotransferase; CAP, controlled attenuation parameter; HS-CRP, high sensitivity C-reactive protein; OR, odds ratio; CI, confidence interval. Adjusted for age, sex, hypertension, body mass index, fasting glucose, cholesterol, triglyceride, high-density lipoprotein cholesterol, ALT and hs-CRP adjusted; * overall *p*-value.

## Data Availability

The data presented in this study are available on request from the corresponding author.

## Supplementary Materials

The following are available online at https://www.mdpi.com/article/10.3390/life11080848/s1, Table S1: Parameters associated with low muscle mass (class II). Table S2: Subgroup analysis of hepatic steatosis in relation with low muscle mass (class I) according to obesity
