# Supplementary material for: The Association between Low Muscle Mass and Hepatic Steatosis in Asymptomatic Population in Korea
Source: Life (Basel). 2021 Aug 19;11(8):848. doi: 10.3390/life11080848 (PMC8400877; doi:10.3390/life11080848)
Supplement: Supplementary file 1 [file life-11-00848-s001.zip › life-1295872-supplementary.pdf]

Supplementary Table 1. Parameters associated with low muscle mass (class II)

Variables	Odds ratio	95% confidence interval	*P*-value*
Age, years	1.019	1.000-1.040	0.071
Male	2.483	1.510-4.084	<0.001
Hypertension	1.902	1.262-2.868	0.002
Diabetes mellitus	1.495	0.873-2.557	0.143
Hypercholesterolemia	1.195	0.776-1.839	0.419
Current smoking	1.080	0.661-1.767	0.758
Body mass index, kg/m^2	1.607	1.476-1.748	<0.001
Fasting glucose, mg/dL	1.011	1.004-1.018	0.004
Total cholesterol, mg/dL	1.006	1.001-1.011	0.015
Triglyceride, mg/dL$^+$	2.607	1.791-3.794	<0.001
HDL cholesterol, mg/dL	0.978	0.963-0.993	0.004
AST, IU/L$^+$	1.355	0.822-2.233	0.233
ALT, IU/L$^+$	2.056	1.432-2.953	<0.001
HS-CRP (mg/dL)	1.947	1.296-2.924	0.001
CAP, dB/m	1.018	1.014-1.023	<0.001
CAP≥248dB/m	4.825	2.855-8.154	<0.001
CAP≥302dB/m	4.645	3.049-7.077	<0.001
LSM, kPa	1.276	1.137-1.432	<0.001
LSM, tertile (1)	1 (reference)		<0.001
tertile (2)	2.118	1.162-3.859	0.014
tertile (3)	3.514	1.986-6.219	<0.001

ALT, alanine aminotransferase; AST, aspartate aminotransferase; CAP, controlled attenuation parameter; HDL, high-density lipoprotein; HS-CRP, high sensitivity C-reactive protein; LSM, liver stiffness measurement. * compared to no sarcopenia as reference, $^+$ log transformed

Supplementary Table 2. Subgroup analysis of hepatic steatosis in relation with low muscle mass (class I) according to obesity.

	Non-obese		Obese	
	OR (95% CI)	*P*-value	OR (95% CI)	*P*-value
CAP≥248 (vs <248) dB/m	2.22 (1.46-3.37)	<0.001	2.04 (1.10-3.78)	0.024
CAP Grade		<0.001*		0.007*
CAP ≥248, <302 (vs <248)dB/m	1.74 (1.05-2.88)	0.033	1.34 (0.67-2.69)	0.407
CAP ≥302 (vs <248) dB/m	2.90 (1.74-4.85)	<0.001	2.95 (1.45-5.98)	0.003
Liver stiffness measurement				
T3 (vs T1)§	2.04 (1.25-3.32)	0.004	2.23 (1.12-4.45)	0.022

ALT, alanine aminotransferase; CAP, controlled attenuation parameter; OR, odds ratio; CI, confidence interval; HS-CRP, high sensitivity C-reactive protein.
Adjusted for age, sex, hypertension, diabetes, hypercholesterolemia, triglyceride, high-density lipoprotein cholesterol, hs-CRP, and ALT. * overall P-value.
§ liver stiffness measurement in tertile, T1 vs. T3.
